# Effect of a weak transverse magnetic field on the microstructure in directionally solidified peritectic alloys

**DOI:** 10.1038/srep37872

**Published:** 2016-11-25

**Authors:** Xi Li, Zhenyuan Lu, Yves Fautrelle, Annie Gagnoud, Rene Moreau, Zhongming Ren

**Affiliations:** 1 Department of Material Science and Engineering, Shanghai University, Shanghai, 200072, P. R. China; 2 EPM-Madylam, ENSHMG BP 38402 St Martin d’Heres Cedex, France

## Abstract

Effect of a weak transverse magnetic field on the microstructures in directionally solidified Fe-Ni and Pb-Bi peritectic alloys has been investigated experimentally. The results indicate that the magnetic field can induce the formation of banded and island-like structures and refine the primary phase in peritectic alloys. The above results are enhanced with increasing magnetic field. Furthermore, electron probe micro analyzer (EPMA) analysis reveals that the magnetic field increases the Ni solute content on one side and enhances the solid solubility in the primary phase in the Fe-Ni alloy. The thermoelectric (TE) power difference at the liquid/solid interface of the Pb-Bi peritectic alloy is measured *in situ*, and the results show that a TE power difference exists at the liquid/solid interface. 3 D numerical simulations for the TE magnetic convection in the liquid are performed, and the results show that a unidirectional TE magnetic convection forms in the liquid near the liquid/solid interface during directional solidification under a transverse magnetic field and that the amplitude of the TE magnetic convection at different scales is different. The TE magnetic convections on the macroscopic interface and the cell/dendrite scales are responsible for the modification of microstructures during directional solidification under a magnetic field.

Peritectic equilibrium is found in many alloys, including the commercially important Fe-C and Fe-Ni alloys and some Al-based alloys^1^. Peritectic alloy solidification involves the nucleation and growth of the primary phase and peritectic phase. A wide variety of possible microstructures can occur, such as alternating banded microstructures between the primary phase and peritectic phase with different interface morphologies, island-like microstructures and simultaneous coupled microstructures, etc.^2^,^3^,^4^. In the past few years, a large number of numerical simulations and hypothesis models have been reported to explain the complex solidification structures in directionally solidified peritectic alloys^5^,^6^. Research results have shown that convection is an important factor in affecting the microstructures in peritectic alloys^7^.

The effect of a magnetic field on convection during the solidification process has always been a research hotspot. Normally, the application of a static magnetic field will dampen the convection. However, Tewari *et al*.^8^ found that freckles formed in a directionally solidified Pb-Sn alloy under a 0.45 T transverse magnetic field at low growth speeds. Alboussiere *et al*.^9^ also found that freckles appeared in a Bi-Sn alloy under a 0.6 T transverse magnetic field and suggested that a new convection was created. In our previous works^10^,^11^, the effect of the magnetic field on the solidification behaviors in single phase alloys and eutectic alloys was investigated. The experimental and numerical results showed that the TE magnetic convection played a key role in affecting the solidification structures during directional solidification under a magnetic field. However, so far, little work has been perfromed to study the effect of a magnetic field on the solidification behaviors of peritectic alloys. Therefore, it is necessary and valuable to study the effect of a magnetic field on the microstructures in peritectic alloys during directional solidification.

The aim of the present work is twofold: on one hand, the effect of a weak transverse magnetic field on the solidification structure in peritectic Fe-Ni and Pb-Bi alloys is investigated; on the other hand, by studying the influence of the magnetic field on the solidification structure in peritectic alloys, an understanding of the effect of convection on the solidification structure in peritectic alloys may be extended and deepened.

## Experimental

Fe-4.2, 4.33 and 4.5 at% Ni and Pb-25 at% Bi alloys were prepared with high purity Fe (99.99 wt.%), Ni (99.999 wt.%), Pb (99.99 wt.%), and Bi (99.999 wt.%) in a vacuum suspension melting furnace. Cast samples were enveloped in tubes of high-purity corundum with an inner diameter of 3 mm and a length of 200 mm for the directional solidification.

A schematic illustration of the experimental apparatus is shown in ref. 12. It consists of a direct current electromagnet, a Bridgman furnace and a growth velocity and temperature controller. The direct current electromagnet can produce a transverse static magnetic field up to 1.0 T. The temperature in the furnace was controlled by a Pt/6Rh-Pt/30Rh thermocouple inserted in a pure alumina tube. During the directional solidification, the specimens in the crucibles were melted and maintained for 30 min, and then solidified in the Bridgman apparatus by pulling the crucible assembly at various velocities with and without a transverse magnetic field. At the end of the experiment, the crucibles were quickly dropped into a Ga-In-Sn metal to obtain the microstructure of the solid/liquid interface.

The obtained specimens were cut along the direction perpendicular to the magnetic field. The transverse section was observed at a position of approximately 5 mm from the solid/liquid interface. The microstructures were examined in the etched condition by optical microscopy. An electron probe micro analyzer (EPMA) was utilized to measure the distribution of the solute, and the corresponding error was approximately 2–3%.

The numerical simulation was performed by using the finite element commerical code COMSOL Multiphysics, available in the laboratory (SIMAP-EPM / G-INP / CNRS). In the present numerical work, the temperature field (*T*), thermoelectric current density (*J*_TE_), fluid flow field (*V*), and the magnetic field intensity (*B*) are coupled. The study configuration is three-dimensional and both the solid and the liquid are considered. The solid/liquid interface is prescribed, and its shape is a simplified representation of the solidification structures. Two types of domain are considered. The first one consists of a curved interface at the scale of the sample, while the second comprises a cell at the scale of the cell/dendrite. The basic equations of the numerical simulation are


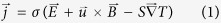



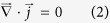



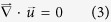


where 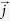 is the electric current density, *σ* is the electrical conductivity, *E* is the electrical field, *u* is the fluid velocity of moving substance in a magnetic field *B*, 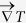 is the temperature gradient and *S* is the thermoelectric power of the material. The temperature gradient is prescribed at infinity


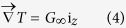


where *G*_*∞*_ is a constant of 60 K/cm, and i_z_ is a unit vector along the z-axis.


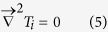


where i = l or s in the liquid or in the solid, respectively. The details of the basic assumptions and boundary conditions are shown in ref. 13.

## Results

**Figure 1:**
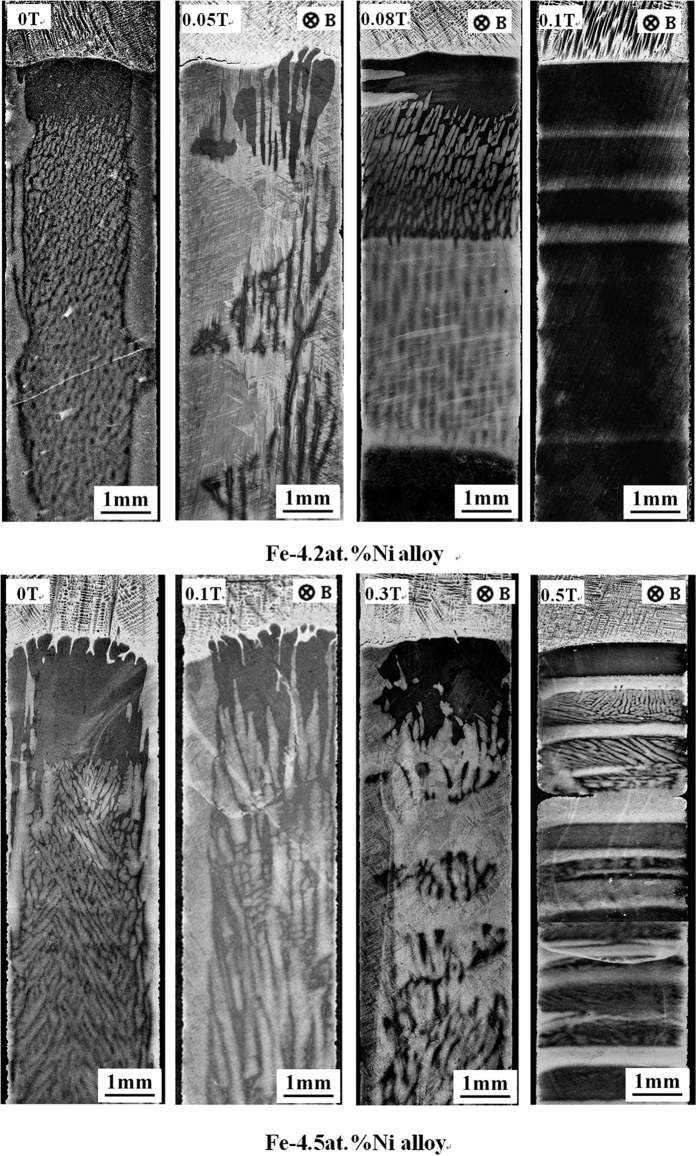
Longitudinal structures near the solid/liquid interface in directionally solidified Fe-Ni peritectic alloys at 2 μm/s under various magnetic fields. Dark phase = α, light phase = β, G_K_ = 60 K/cm.

**Figure 2:**
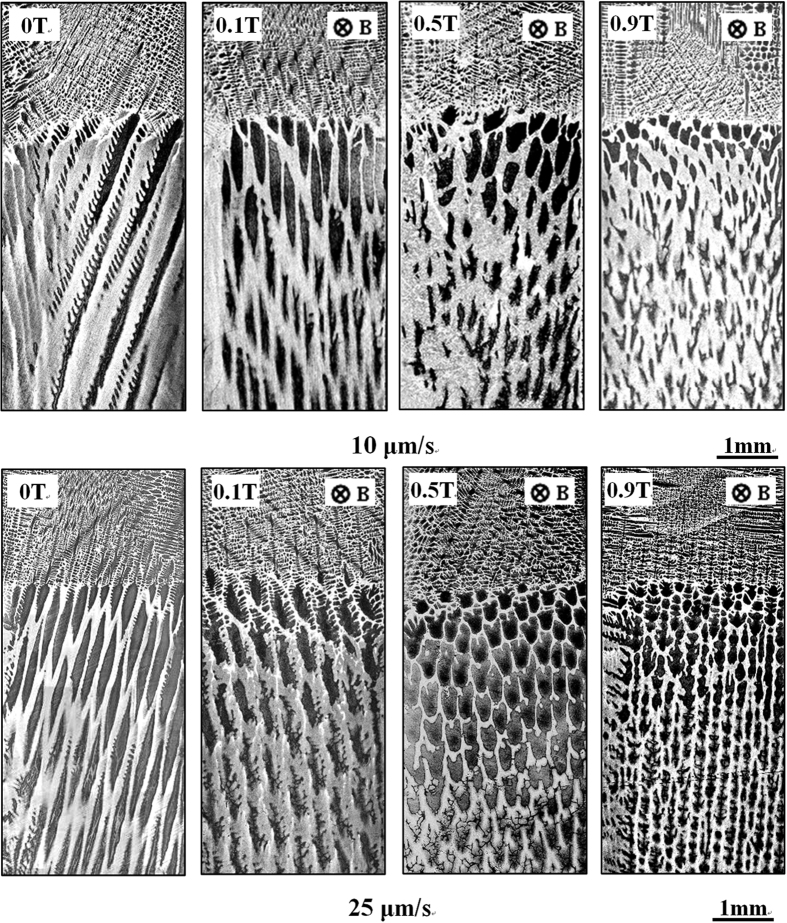
Longitudinal structures near the solid/liquid interface in directionally solidified Fe-4.2 at.%Ni hypoperitectic alloy at the growth speeds of 10 μm/s and 25 μm/s under various magnetic fields. Dark phase = α, light phase = β, G_K_ = 60 K/cm.

**Figure 3:**
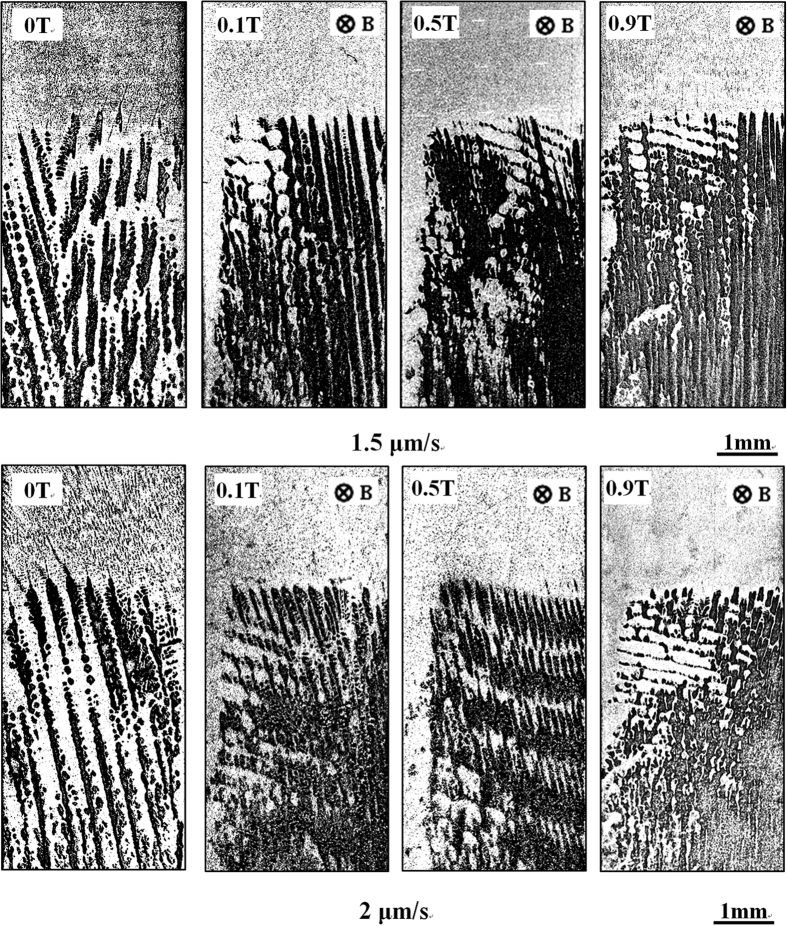
Longitudinal structures near the liquid/solid interface in directionally solidified Pb-25at.%Bi hypoperitectic alloy at the growth speeds of 1.5 μm/s and 2 μm under various magnetic fields. Dark phase = α, light phase = β, G_K_ = 80 K/cm.

Figure 1 shows the longitudinal microstructures near the solid/liquid interface in directionally solidified Fe-4.2 at.%Ni hypoperitectic and Fe-4.5 at.%Ni hyperperitectic alloys at a growth speed of 2 μm/s under various magnetic fields. The microstructure consists of a primary α-phase (black) and a peritectic β-phase (gray). For the Fe-4.2 at.%Ni and Fe-4.5 at.%Ni alloys, the primary α-phase is the ferrite, and the peritectic β-phase is the austenite. In the case of no magnetic field, the solid/liquid interface is slightly convex near the edges owing to some lateral temperature gradient. Ni enrichment by macrosegregation is another cause for that observation^14^. The morphology of the solid/liquid interface is planar and cellular structures of the primary α-phase in the central region and island-like structures of the peritectic β-phase at the edge. When a transverse magnetic field is applied, the shape of the liquid/solid interface becomes wavy, and the peritectic β-phase appears at one edge of the sample under a lower magnetic field. With the increase in the magnetic field, the shape of the liquid/solid interface becomes flat and the peritectic β-phase grows along the lateral direction. Finally, alternate banded structures of α- and β-phases form in the Fe-4.2 at.%Ni alloy under a 0.1 T magnetic field and in the Fe-4.5 at.%Ni alloy under a 0.5 T magnetic field. Figure 2 shows the longitudinal structures near the solid/liquid interface in directionally solidified Fe-4.2 at.%Ni hypoperitectic alloy at growth speeds of 10 μm/s and 25 μm/s under various magnetic fields. In the case of no magnetic field, the structure is cellular/dendritic. When the magnetic field is applied, the structures transform into island-like structures, and the size of the island-like band decreases with the increase in the magnetic field. Figure 3 shows the longitudinal structures near the liquid/solid interface in directionally solidified Pb-25at.%Bi hypoperitectic alloy at growth speeds of 1.5 μm/s and 2 μm/s under various magnetic fields. When the magnetic field is applied, a single peritectic β-phase forms on one side of the sample and island-like structures appear near the liquid/solid interface. The above results are enhanced as the magnetic field increases.

**Figure 4:**
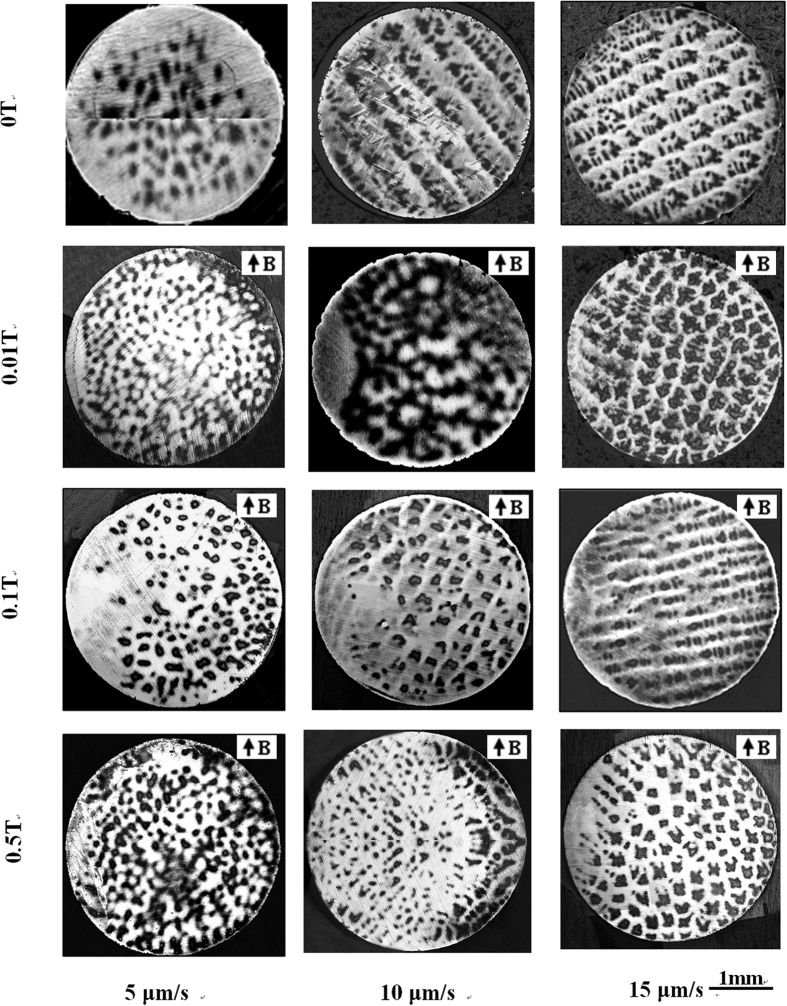
Transverse structures at 5 mm from the liquid/solid interface in directionally solidified Fe-4.2 at.%Ni hypo-peritectic alloy at a certain growth speed under various magnetic fields. Dark phase = α, light phase = β, G_K_ = 60 K/cm.

**Figure 5:**
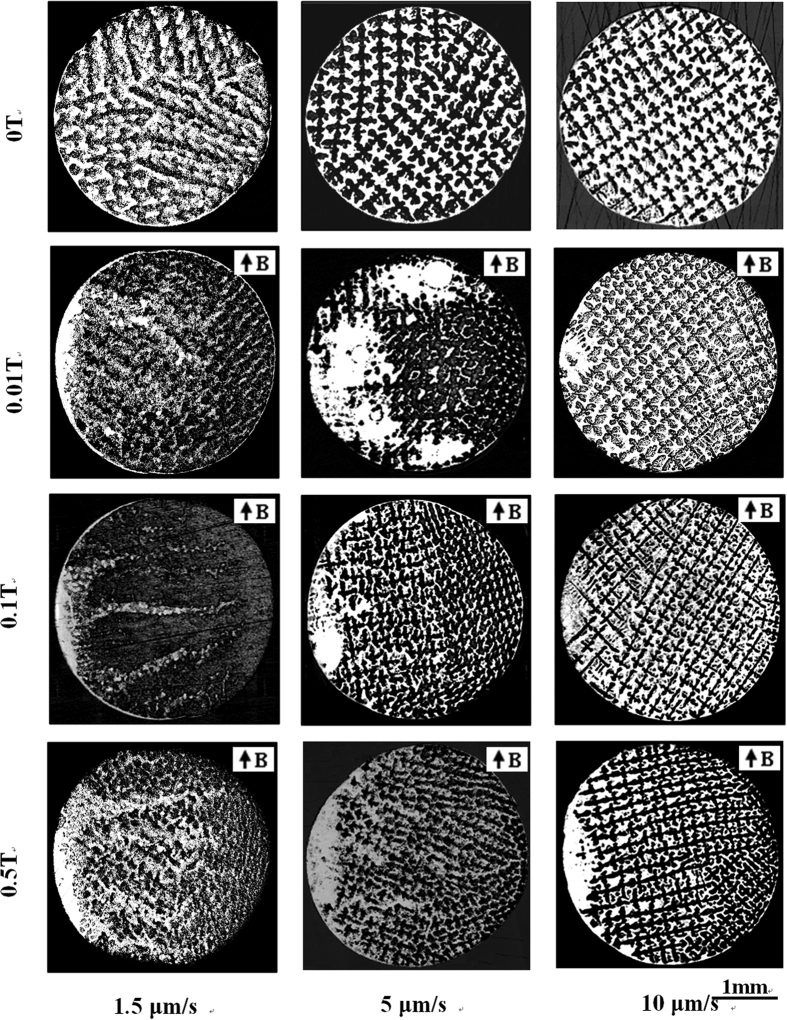
Transverse structures at 5 mm from the liquid/solid interface in directionally solidified Pb-25at.%Bi hypo-peritectic alloy at a certain growth speed under various magnetic fields. Dark phase = α, light phase = β, G_K_ = 80 K/cm.

**Figure 6:**
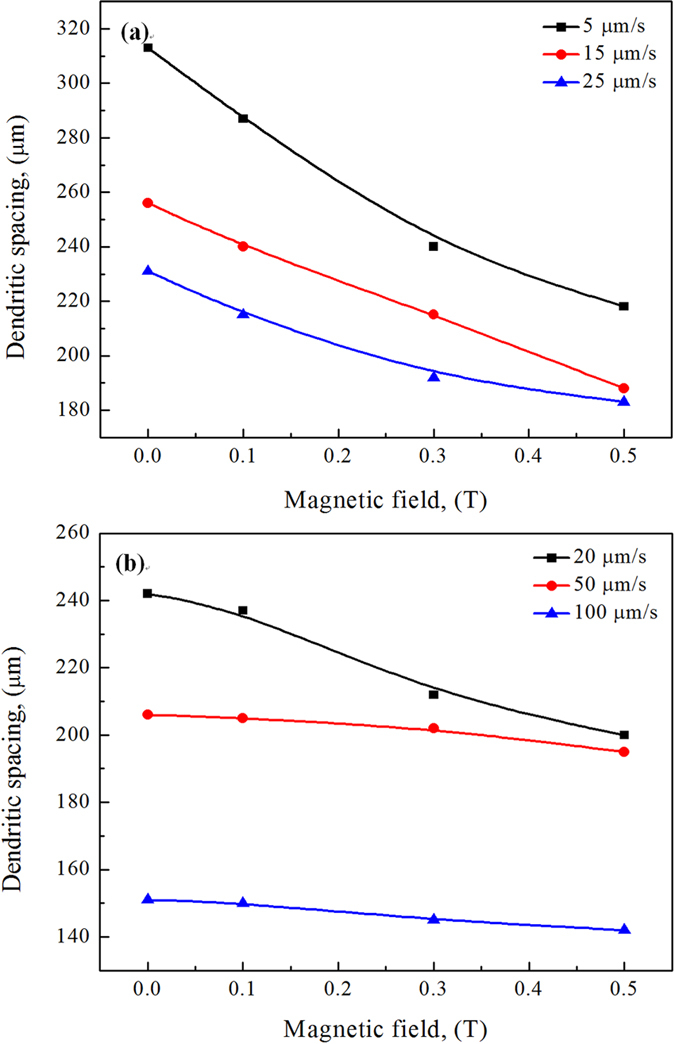
Effect of the magnetic field on the dendritic/cellular spacing of the primary phase during directional solidification of Fe-4.5 at.%Ni alloy (**a**) and Pb-25at.% Bi alloy (**b**).

Figures 4 and 5 show the transverse microstructures in directionally solidified Fe-4.2 at.%Ni and Pb-25 at.%Bi alloys, respectively. It can be observed that the application of the magnetic field causes the single β-peritectic phase to appear on one side of the sample, and the size of the cell/dendrite to decrease during directional solidification. The cellular/dendritic spacing is measured and the results are shown in Fig. 6. The magnetic field decreases the cellular/dendritic spacing and this effect weakens with the increase of the growth speed.

**Figure 7:**
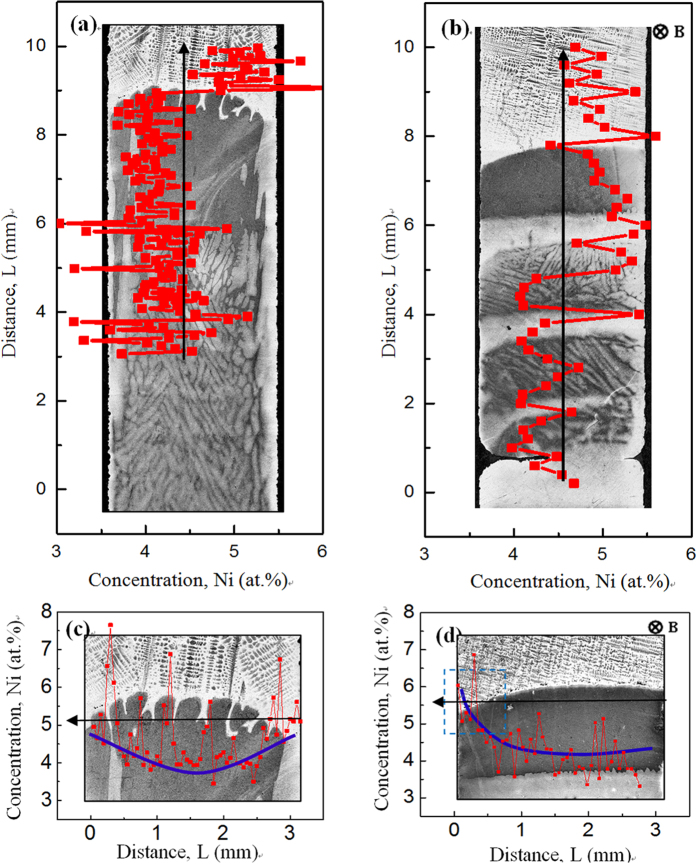
EPMA analysis for the distribution of the Ni solute in directionally solidified Fe-4.5 at.%Ni hyperperitectic alloy at 2 μm/s without and with a 0.5 T magnetic field: (**a**,**b**) Showing the distribution of the Ni solute along an axial direction without and with the magnetic field, respectively; (**c**,**d**) showing the distribution of the Ni solute along radial direction without and with the magnetic field, respectively. The blue line shows the schematic illustrating of the distribution of the Ni solute.

**Figure 8:**
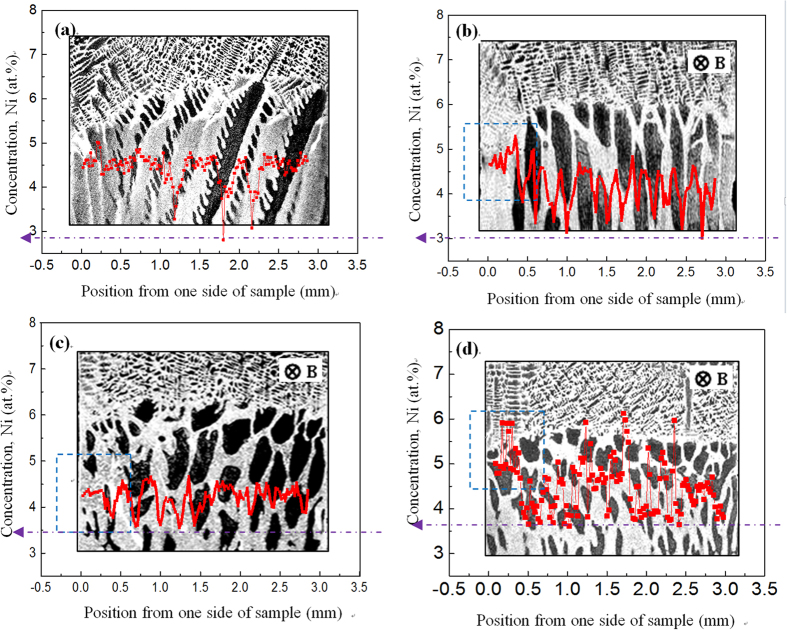
Radial distribution of the Ni solute in the solid at 300 μm from the liquid/solid interface in directionally solidified Fe-4.2 at.%Ni hypoperitectic alloy at 10 μm/s under various magnetic fields: (**a**) 0 T; (**b**) 0.1 T; (**c**) 0.5 T; (**d**) 0.9 T.

Furthermore, EPMA is used to measure the distribution of the Ni solute content on the scales of the sample and cell/dendrite. Figure 7 shows the axial and radial distributions of the Ni solute content in Fe-4.5 at.%Ni hyperperitectic alloy directionally solidified at a growth speed of 2 μm/s without and with a 0.5 T magnetic field. In the case of no magnetic field, the concentration of the Ni solute at the edge is higher than that in the central region (see Fig. 7(c)). The blue line shows the schematic illustrating of the distribution of the Ni solute. Moreover, the magnetic field causes a periodic change in the Ni solute content along the axial direction (see Fig. 7(b)) and an increase in the Ni solute content on one side of the sample (see Fig. 7(d)). Figure 8 shows the radial distribution of the Ni solute content in Fe-4.2 at.%Ni hypoperitectic alloy directionally solidified at a growth speed of 10 μm/s under various magnetic fields. One can notice that the distribution of the Ni solute content shows periodic changes, and this distribution period becomes short and irregular under the magnetic field. It is also found that the minimum value of the Ni content in the primary α-phase increases as the magnetic field increases. This implies that the application of the magnetic field causes an increase in the solid solubility in the primary α-phase during directional solidification.

## Discussion

### TE magnetic convection during directional solidification of peritectic alloys under a transverse magnetic field

**Figure 9:**
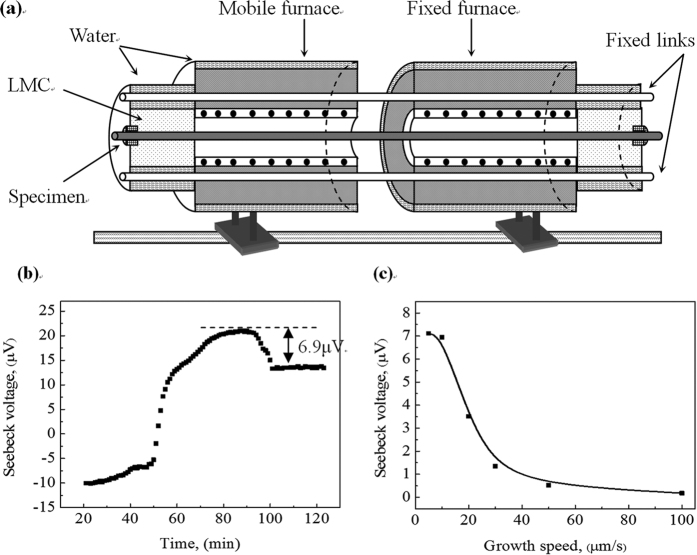
(**a**) Schematic illustration of the measuring apparatus for the Seebeck voltage; (**b**) Seebeck voltage as a function of time in the Pb-25 at.% Bi alloy directionally solidified at 10 μm/s; (**c**) Seebeck voltage as a function of the growth speed in the Pb-25 at.% Bi peritectic alloy.

As is well known, convection can significantly affect the solidification structure in peritectic alloys during directional solidification^7^. A previous work has proven that a TE magnetic convection will be produced during directional solidification under a magnetic field^15^. Owing to the Seebeck effect, in any material, a temperature gradient 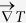 produces a Seebeck electromotive force 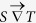, where *S* is the thermoelectric power of the material^16^. If two metals with different TE power, *S*_*S*_ and *S*_*L*_ (*S*_*S*_ is the TE power of the solid, and *S*_*L*_ is the TE power of the liquid), joined in two places with a temperature gradient between the joints, then a TE current is generated in the system. Thus, the interaction between the TE current and the magnetic field will produce a TE magnetic convection at the vicinity of the interface and the corresponding recirculation loops will form at the crucible scale. To confirm the existence of the TE power difference at the liquid/solid interface (*η*_*sl*_ = *S*_*s*_ − *S*_*L*_) during directional solidification of the peritectic alloys, the Seebeck signals of the Pb-Bi peritectic alloy are measured *in situ*. The basic principle of the interfacial TE measurement using the Seebeck technique is schematically presented in Fig. 9(a). It consists of two furnaces each with a neighboring heat sink that is cooled by the Ga-In-Sn metal. One of the furnaces is fixed, while the other can be moved at a given velocity on a pulling platform. Therefore, two solid/liquid interfaces are created and linked together with the liquid phase in the sample during directional solidification. Due to the Seebeck effect, the Seebeck voltage will be measured between the both ends of the sample. The details of the principle are shown in refs 17 and 18. Figure 9(b) shows the Seebeck response obtained for the Pb-25 at.% Bi alloy at a growth speed of 10 μm/s. One can see that a plateau is obtained on the Seebeck curve, which indicates the establishment of a steady state, and then the signal decreases to a new steady state when the furnace translation is shut off. The magnitude of the signal drop, *E*_*s*_ = (*S*_*S*_ − *S*_*L*_)Δ*T*, corresponds directly to the effective liquid/solid TE power difference (*η*_*sl*_). Figure 9(c) shows the Seebeck voltage as a function of the growth speed. One can see that the Seebeck voltage decreases with the increase in the growth speed, and then the change in the Seebeck voltage weakens as the growth speed continues to increase.

**Figure 10:**
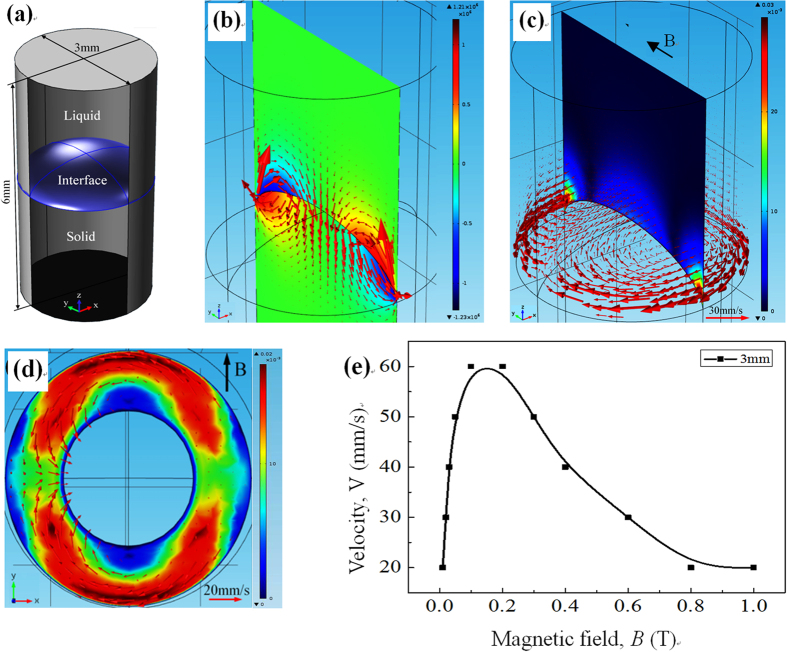
(**a**) 3 D geometry used for the numerical simulation of the TE magnetic effects at the liquid/solid interface in the Fe-Ni alloy; (**b**) general 3 D view of the TE current, (**c**,**d**) general view of the computed TE magnetic convection in the liquid near the interface and corresponding 2 D view seen from positive z-axis under the 0.05 T transverse magnetic field and a temperature gradient of 60 K/cm, respectively; (**e**) velocity of the TE magnetic convection as a function of the magnetic field. Red arrows present flow field, the color slice is the magnitude.

**Figure 11:**
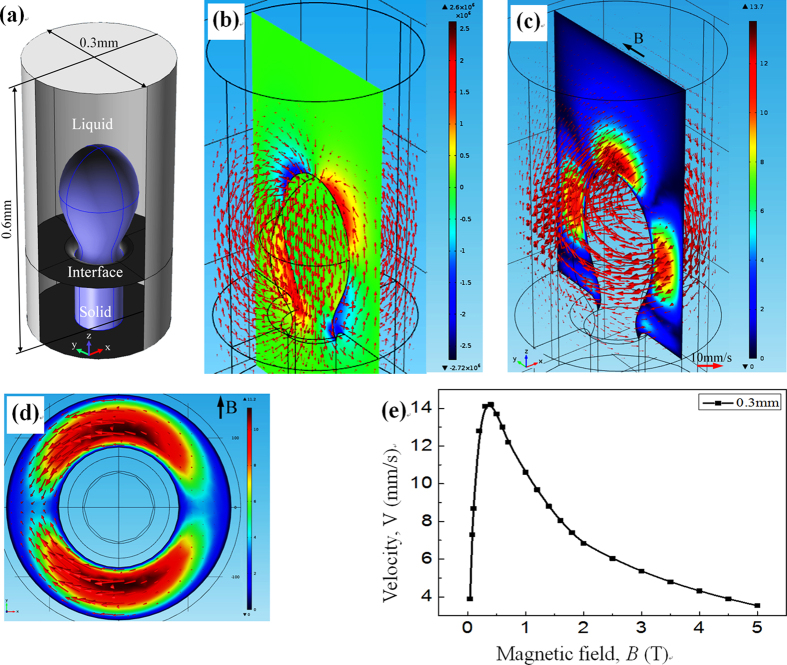
(**a**) 3 D geometry used for the numerical simulation of the TE magnetic convection in the liquid between cells/dendrites in the Fe-Ni alloy. (**b**) general 3 D view of the TE current; (**c**,**d**) general 3 D view of the TE magnetic convection in the liquid near the interface and corresponding 2 D view seen from positive z-axis under the 0.05 T transverse magnetic field and a temperature gradient of 60 K/cm, respectively; (**e**) the velocity of the TE magnetic convection as a function of the magnetic field. Red arrows present flow field, the color slice is the magnitude.

Furthermore, to study the amplitude and distribution of the TE magnetic convection during directional solidification of peritectic alloys under a transverse static magnetic field, a 3 D numerical simulation for the Fe-Ni alloy is performed. Figures 10(a) and 11(a) show the 3 D geometry at the liquid/solid interface and between cells used for the simulation of the TE magnetic effects. Table 1 shows the physical properties of the Fe-Ni alloy used during the numerical simulation process. During the calculation, to avoid affecting the flows, the viscosity for the solid phase was at 4 × 10^3^ Pa·s. Figures 10(b) and 11(b) respectively show the typical computed TE currents in the liquid near the liquid/solid interface and between the cells. One can notice that the TE current forms the circuits along the liquid/solid interface and the cell, and the TE current has the most intense densities in the region near the interface. Figure 10(c,d) respectively show the general 3-D view of the computed TE magnetic convection and the corresponding 2-D view seen from the positive z-axis under a transverse magnetic field of 0.05 T and a temperature gradient of 60 K/cm. The TE magnetic convection flows from the edge of the sample and returns to the region higher in bulk, and the maximum of the convection appears at the edge of the sample perpendicular to the magnetic field. Figure 11(c,d) respectively show the general 3-D view of the computed TE magnetic convection in the liquid between cells and the corresponding 2-D view seen from the positive z-axis under a transverse magnetic field of 0.05 T and a temperature gradient of 60 K/cm. The convection flows from the mushy zone and returns to the region higher in bulk. Note that the TE magnetic convection is unidirectional from positive x-axis to the negative, and then through the region ahead of the cell. Figures 10(e) and 11(e) respectively show the values of the TE magnetic convection in the liquid at the liquid/solid interface and between the cell/dendrite as a function of the magnetic field. One can see that the velocity of the TE magnetic convection increases and reaches a maximum value with the increase in the magnetic field intensity, and then the value of the TE magnetic convection decreases as the magnetic field intensity continue to increase. The magnetic fields corresponding to the maximum value of the TE magnetic convection at the sample and cell scales are approximately 0.1 T and 0. 5 T, respectively.

### Transverse magnetic field-induced formation of banded structure

**Figure 12:**
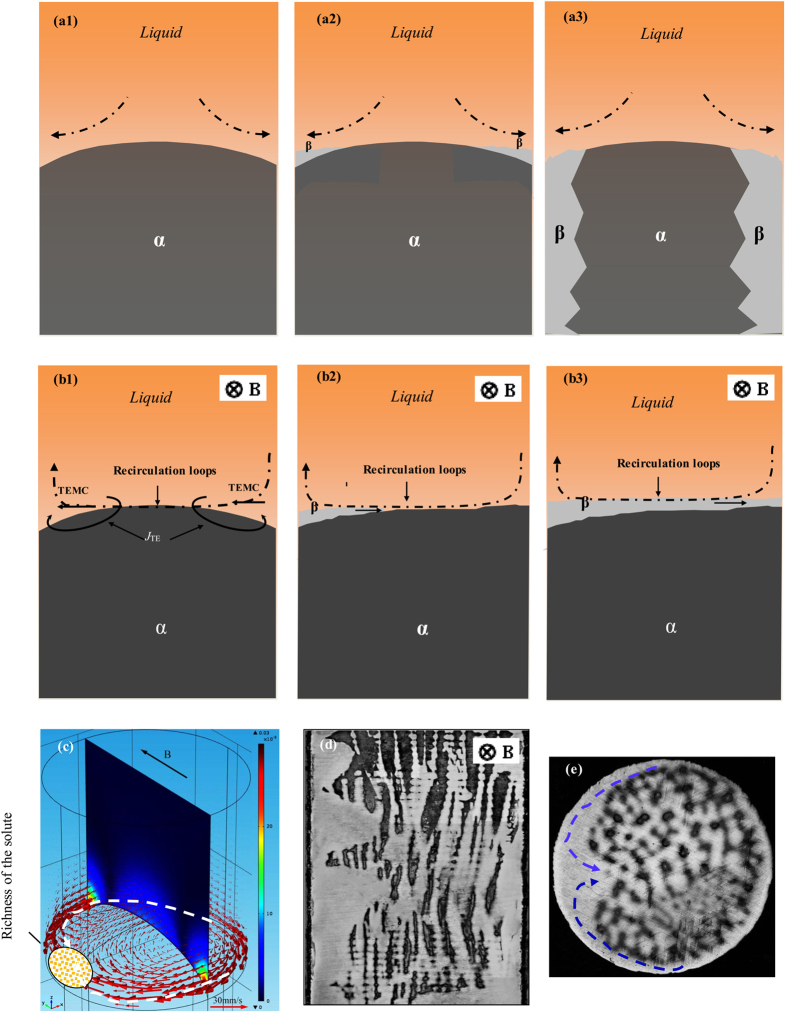
Schematic illustration of the convections in the liquid near the solid/liquid interface with and without the magnetic field and the influences of the convection on solidification structure during directional solidification of peritectic alloys: (a1–a3) Natural convection in the liquid near the solid/liquid interface and the formation of island-like structure at the edge of the sample in the case of no magnetic field; (b1–b3) the TE magnetic convection in the liquid near the solid/liquid interface and the formation of banded structure under the TE magnetic convection; (**c**) typical computed TE magnetic convection during directional solidification under a transverse magnetic field; (**d**) and (**e**) showing longitudinal and transverse structures in directionally solidified Fe-4.2 at.%Ni peritectic alloys, respectively (R = 5 μm/s, B = 0.1 T).

The above experimental results reveal that the application of a transverse magnetic field during directional solidification causes the formation of a banded structure in Fe-Ni samples (see Fig. 1) and a single peritectic phase region at one edge of the Fe-Ni and Pb-Bi samples. This may be attributed to the effect of TE magnetic convection at the sample scale on the distribution of the solute under a transverse magnetic field. Generally, for vertical Bridgman crystal growth of Fe-Ni alloys with the melt above the crystal, the heavier Ni species migrate down to the protruding liquid/solid interface due to gravitational force, as shown in Fig. 12(a). Because the solidification temperature decreases as the local concentration of the heavier Ni species increases, the solidification temperature for a Ni-rich melt at the ampoule wall is lower than that for a Fe-rich melt at the centerline, leading to a liquid/solid interface, which is far more protruding than the local isothermal surface, as shown in Fig. 12(a). When the content of the Ni solute reaches a certain value, the peritectic β-phase will nucleate and grow at the edge of the sample. The solute enriches in front of the liquid/solid interface^19^ and the discrete crystal growth^20^,^21^,^22^,^23^ has been focused and investigated. Under the magnetic field, TE magnetic convection will form in the liquid near the liquid/solid interface during directional solidification. At the same time, the TE magnetic convection will induce a loop solute flow in the liquid in front of the solid/liquid interface, as shown in Fig. 12(b1). These convections will cause the Ni solute to migrate and gather on one side of the specimen, as shown in Figs 10(d) and 12(b1). When the content of the Ni solute reaches a certain value, the peritectic phase will nucleate at the liquid/solid/wall junction and grow along the lateral direction. As the TE magnetic convection can provide solute to feed the peritectic β-phase, the primary phase will be covered completely by the peritectic β-phase, as shown in Fig. 12(b3). Because the peritectic β-phase rejects less solute than the primary α-phase, the magnitude of the solute boundary layer ahead of the liquid/solid interface decreases, and consequently, the interface temperature increases. Above the peritectic temperature, the primary α-phase re-nucleates on the peritectic β-phase at another nucleation undercooling. The whole cycle then repeats itself, leading to the formation of alternate solid α- and β-layers. From Fig. 10, it can be observed that the value of the TE magnetic convection at the sample scale increases with increasing magnetic field when the applied magnetic field is less than 0.1 T. Therefore, the formation of the banded structure is enhanced with the increase in the magnetic field when the applied magnetic field is less than 0.1 T.

### Magnetic field-induced formation of island-like structure and refinement of the primary phase

The above results also reveal that a magnetic field causes the formation of an island-like structure and modifies the cell/dendrite spacing in the peritectic alloys during directional solidification. This may be attributed to the TE magnetic convection at the cell/dendrite scale.

**Figure 13:**
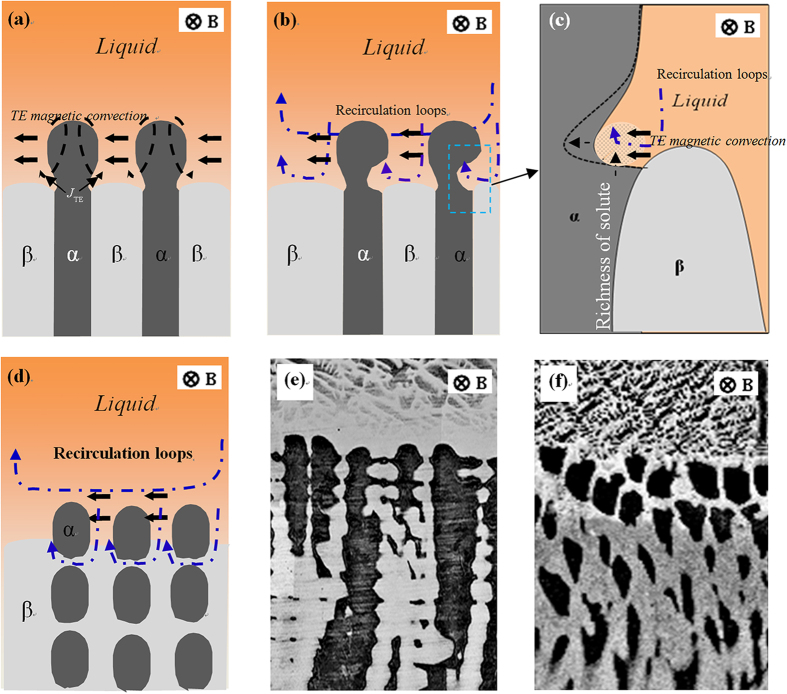
Schematic illustration of the TE magnetic convections in the liquid between cells/dendrites under the transverse magnetic field and the influences of the convections on the cell/dendrite morphology during directional solidification of peritectic alloys: (**a**) TE magnetic convection in the liquid between cell/dendrite; (**b**–**d**) the effect of the TE magnetic convection and corresponding recirculation loops on the cell morphology; (**e**,**f**) the evolution of the cell morphology during directional solidification of the Fe-Ni peritectic alloys under the transverse magnetic field.

As shown in Fig. 13(a), when a magnetic field is applied during directional solidification, TE magnetic convection will be produced in the liquid between cells/dendrites, and then recirculation loops are induced, as shown in Fig. 13(b). The TE magnetic convection will cause an enrichment of the solute at the liquid/α/β tri-junctions, as shown in Fig. 13(c). As a result, the primary α-phase may be remelted and transformed into discrete islands, as shown in Fig. 13(d–f). Moreover, the convection at the cell/dendrite scale will enhance the diffusion of the solute to the primary phase and cause the increase in the solid solubility in the primary phase. Because the velocities of the TE magnetic convection at the cell/dendrite scale increase with increasing magnetic field intensity when the magnetic field is lower than 0.5 T, the island-like structures are enhanced with increasing magnetic field under a lower magnetic field.

Lehmann *et al*.^24^ have studied the effect of convection on the cellular/dendritic spacing, with the expression 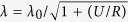, where *λ*_*0*_ is the primary spacing without convection, *U* is the convection velocity and *R* is the growth speed. According to the above equation, convection will reduce the cell/dendrite spacing. From the above numerical results, it can be seen that the velocities of the TE magnetic convection increase with the continuous increase in the magnetic field under a weak transverse magnetic field. This is an agreement with the change in the cellular/dendritic spacing under the magnetic field. Therefore, the change in the cellular/dendritic spacing under the magnetic field should be attributed to the TE magnetic convection at the cell/dendrite scale.

## Conclusion

In summary, by directional solidification experiments with Fe-Ni and Pb-Bi peritectic alloys under a weak transverse magnetic field, it is found that the magnetic field can induce the formation of banded and island-like structures and refine the primary phase in the peritectic alloys. Further, EPMA and microscopy analyses reveal that the concentration of the Ni solute increases on one side of the sample and the solid solubility in the primary phase is enhanced under the magnetic field. The numerical results show that a unidirectional TE magnetic convection forms in the liquid near the liquid/solid interface and that the TE magnetic convection at different scales was different during directional solidification under a transverse magnetic field. Consequently, the changes in the solute distribution, induced by the TE magnetic convection under the magnetic field, should be responsible for the formation of banded structures and island-like structures in the peritectic alloys. Specifically, the formation of the banded structures should be attributed to the TE magnetic convection at the scale of the macroscopic interface, and the formation of the island-like structures and the refinement of the primary phase should be attributed to the TE magnetic convection at the scale of the cell/dendrite.

## Additional Information

**How to cite this article**: Li, X. *et al*. Effect of a weak transverse magnetic field on the microstructure in directionally solidified peritectic alloys. *Sci. Rep.* **6**, 37872; doi: 10.1038/srep37872 (2016).

**Publisher's note:** Springer Nature remains neutral with regard to jurisdictional claims in published maps and institutional affiliations.

## Figures and Tables

**Table 1:** Physical properties of Fe-Ni alloys used in numerical simulation.

Thermal conductivity, k [W/(m*K)]	34.6 (1000 °C)	20 (1600 °C)
Density, ρ [kg/m^3^]	7874 (25 °C)	6980 (1538 °C)
Heat capacity, C_P_ [J/(kg*K)]	460	460
Dynamic viscosity, mu [Pa*s]	4 * 10^3^	5 * 10^−3^
Electrical conductivity, σ [S/m]	10^7^ (1000 °C)	14 * 10^7^ (1550 °C)
Thermoelectric power, S [V/K]	−10^−6^ (1480 °C)	−4 * 10^−6^ (1567 °C)
Temperature gradient, G [K/cm]	60	
